# Insights into MLS_B_ resistance in invasive group A streptococci in West Virginia, USA

**DOI:** 10.1093/jacamr/dlaf021

**Published:** 2025-02-19

**Authors:** Lillie M Powell, Soo Jeon Choi, P Rocco LaSala, Slawomir Lukomski

**Affiliations:** Department of Microbiology, Immunology, and Cell Biology, West Virginia University School of Medicine, 2095 Health Sciences North, Morgantown, WV, USA; Department of Microbiology, Immunology, and Cell Biology, West Virginia University School of Medicine, 2095 Health Sciences North, Morgantown, WV, USA; Department of Pathology, West Virginia University School of Medicine, Morgantown, WV, USA; Department of Microbiology, Immunology, and Cell Biology, West Virginia University School of Medicine, 2095 Health Sciences North, Morgantown, WV, USA

Sir,

In the United States, rates of invasive MLS_B_ (macrolide, lincosamide, streptogramin B) resistant group A *Streptococcus* (iGAS) infections have been on the rise since 2010. This led the Centers for Disease Control (CDC) to designate erythromycin resistant iGAS, which are comprised predominately of strains expressing Erm methyltransferases in USA, as a concerning threat.^1,2^ Cluster analysis of US iGAS isolates revealed that intravenous drug users (IVDUs) are at significant risk for infection with resistant, genomically related isolates.^3^ The state of West Virginia (WV) has the highest rate of opioid related deaths in the nation^4^ but is not part of the CDC surveillance territory. To close this knowledge gap, we previously investigated 133 iGAS isolates collected from a non-paediatric cohort in WV to examine strain diversity and antimicrobial resistance.^5,6^ Here, we further explore genotypic determinants of iGAS MLS_B_ sub-phenotypes, through analysis of the regulatory *erm*-gene promoter.

**Figure 1.**
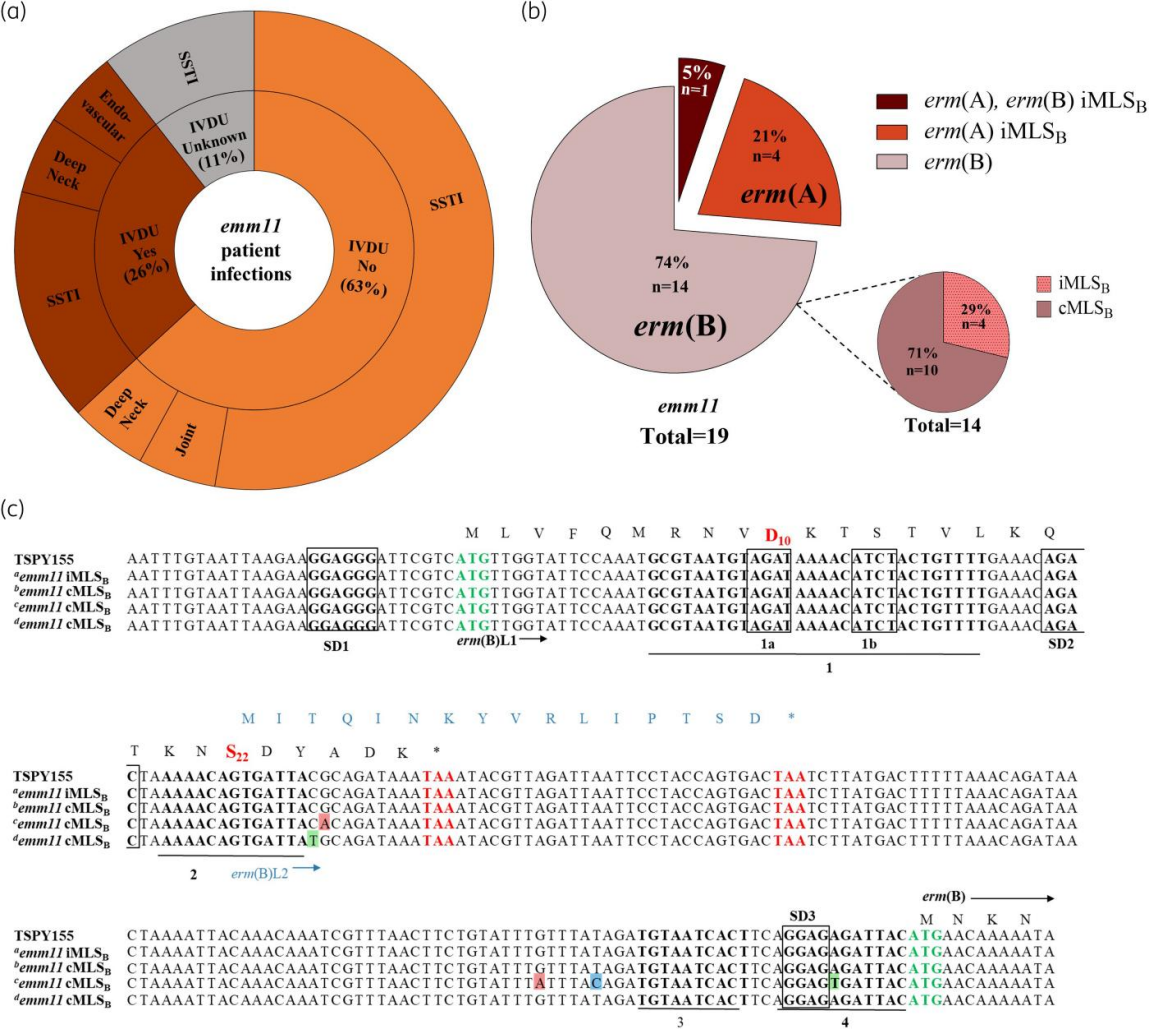
Infection source, MLS_B_ resistance determinants, and *erm*(B) promoter sequence of WV *emm11* isolates. (a) Distribution of intravenous drug use (IVDU) history and source of infections for the 19 *emm11* WV-iGAS isolates. Isolates were recovered consecutively between January 2020-2023, from specimens collected at WVUMedicine system facilities, which are located across the state of WV and in several neighbouring western Maryland, southwestern Pennsylvania, and eastern Ohio counties [the vast majority (82%) of isolates were collected from WV patients]. (b) Percentage of *emm11* WV-iGAS isolates detected by PCR to carry *erm*(A), *erm*(B), or both genes; iMLS_B_ and cMLS_B_  *erm*(B) sub-phenotypes are shown. (c) MAFFT multiple sequence alignment of the *erm*(B) promoter amplified by PCR and sequenced with the Sanger method. Sequences shown are from the top: *erm*(B) regulatory region of *emm11* isolate TSPY155 (accession: CP032699), iMLS_B_ isolate (^a^*n* = 4), cMLS_B_ isolates with no mutations (^b^*n* = 8) and with mutations (^c,d^*n* = 2). The ErmBL1 peptide sequence is shown in black text, whereas the ErmBL2 peptide sequence is shown in blue text. The critical D_10_ and S_22_ amino acids of the ErmBL1 peptide are shown in bold font (red). All Shine Dalgarno sequences are boxed, with the start codon annotated by bold font (green) and the *erm*(B)L stop codons in bold font (red).

In our collection, the two most prevalent WV-iGAS *emm*-types identified were *emm92* (*n* = 61, 46%) and *emm11* (*n* = 19, 14%),^5,6^ whereas the historically and nationally dominant *emm1* iGAS strains were less common (*n* = 3, 2%) and were uniformly MLS_B_ susceptible. Distinct from *emm92*-type iGAS, which is strongly associated with IVDU, CDC surveillance did not find there to be a statistically significant association for *emm11* infection.^1^ In our study, 26% of patients with *emm11* infections and 62% of patients with *emm92* iGAS infections reported a history of IVDU. For either strain *emm* type, skin and soft tissue infections (SSTI) accounted for the vast majority of disease manifestations (Figure 1a).

All *emm11* and *emm92* isolates in our collections displayed MLS_B_ resistance by routine susceptibility testing performed according to CLSI standards.^7^ While isolation of plasmid DNA and PCR amplification revealed that *emm92* isolates uniformly harboured a plasmid-borne *erm*(T) gene, *emm11* isolates harboured chromosomally encoded mobile elements containing *erm*(A) and/or *erm*(B) genes, including 14 (74%) with *erm*(B), 4 (21%) with *erm*(A), and a single isolate (5%) carried both *erm*(A) and *erm*(B) (Figure 1b). Using the conventional D-test to assign the clindamycin resistance sub-phenotype as constitutive (cMLS_B_) or inducible (iMLS_B_) by erythromycin, we found that all *emm11* isolates (*n* = 5) harbouring *erm*(A) (including the *erm*(A) *erm*(B) isolate) exhibited the iMLS_B_ phenotype, which is a similar trend displayed by 87% of *emm92* isolates with *erm*(T)-mediated resistance.^8^ By contrast, the majority (*n* = 10, 71%) of *emm11* isolates with *erm*(B)-mediated resistance exhibited a cMLS_B_ sub-phenotype (Figure 1b), prompting further investigation of the responsible genotype.

The *erm*-gene promoters encode leader peptides (*erm*L) and harbour four inverted repeats (IR) engaged in the formation of two mRNA-hairpin structures. It has been reported that the *erm*L acts in concert with IR1-IR2 and IR3-IR4 hairpins to regulate the expression of their corresponding Erm-methyltransferase enzymes, dependent on the presence of an MLS_B_ antibiotic bound within the ribosomal peptide exit tunnel. The *erm*(A)L in *emm11* and *erm*(T)L in *emm92* iGAS each encodes a single leader peptide with the IFVI-motif that is responsible for ribosome stalling in the antibiotic bound state. Stalling permits the formation of a single alternative IR2-IR3 pairing, thereby releasing the *erm*(A/T) Shine Dalgarno (SD) sequence from the prior IR3-IR4 hairpin, resulting in Erm protein translation.

The regulation of *erm*(B) expression involves two distinct leader peptides *erm*(B)L1/L2 (Figure 1c). In the antibiotic-free non-induced state, two tandem IR1-IR2 and IR3-IR4 hairpins are formed to sequester the *erm*(B)-SD3 sequence within the distal hairpin. In the antibiotic-bound induced state, ribosome stalling occurs at the MRNVD_10_ residue within *erm*(B)L1, which in combination with a frameshift at residue S_22_, results in ErmBL2 synthesis and the formation of the alternative IR2-IR3 hairpin. This conformation allows SD3-mediated translation of the ErmB enzyme.^9^ To test the hypothesis that a cMLS_B_ phenotype results from mutations in the promoter region of the *erm*(B) gene, we sequenced this regulatory region for all cMLS_B_ and iMLS_B_  *emm11* isolates. Of the ten *erm*(B) isolates with constitutive clindamycin resistance by D-test, eight cMLS_B_ isolates showed no mutations compared with iMLS_B_ (Figure 1c, ^a,b^*emm11*), one had four point mutations (^c^*emm11* cMLS_B_) that could favor formation of the hairpin IR2-IR3, and one (^d^*emm11* cMLS_B_) had a single C → T substitution resulting in a missense mutation within the *erm*(B)L2 peptide.

Our data corroborates an earlier report that the cMLS_B_ phenotype is not invariably associated with mutations in the *erm*(B) promoter,^10^ indicating that the regulation of *erm*(B) expression and its loss of inducibility are not solely attributable to leader peptides and mRNA-hairpin structures. In addition, our previous study identified a cMLS_B_  *emm92*-iGAS isolate with no polymorphisms in the *erm*(T) gene promoter,^8^ together advocating for the existence of alternative regulatory mechanism(s) predominately found in the *erm*(B) harbouring strains. Considering that a cMLS_B_ phenotype was associated with an increased clindamycin MIC, and substantial ribosomal methylation in *emm92* in iGAS,^8^ additional regulatory mechanism(s) should be investigated to better understand what effect(s) *erm*-mediated resistance may impart on strain virulence.
